# Inverse association between obesity and intestinal endometriosis: Findings from a cross‐sectional study

**DOI:** 10.1002/ijgo.70930

**Published:** 2026-03-10

**Authors:** Tereza Carolina Fonseca Corrêa, Marco Aurelio Pinho Oliveira

**Affiliations:** ^1^ Department of Gynecology, Pedro Ernesto University Hospital State University of Rio de Janeiro Rio de Janeiro Brazil

**Keywords:** body mass index, body weight, bowel endometriosis, deep endometriosis, endometriosis, intestinal endometriosis, obesity

## Abstract

**Objective:**

This study evaluates the association between obesity and the presence of bowel endometriosis in patients undergoing surgery.

**Methods:**

A retrospective cross‐sectional observational study was conducted with patients treated between September 2023 and December 2024, at a specialized endometriosis outpatient clinic of a tertiary hospital in Rio de Janeiro, Brazil. Women who underwent surgery for histopathological confirmation of deep endometriosis were included. The outcome was bowel endometriosis, defined as a lesion >1 cm located in the rectosigmoid region. Preoperative weight and height were measured, and obesity was defined according to World Health Organization body mass index (BMI) criteria. The association between obesity and the outcome was assessed using multivariable logistic regression, adjusting for potential confounders. A *P*‐value <0.05 was considered statistically significant.

**Results:**

A total of 223 women with histologically confirmed deep endometriosis were included, of whom 24.2% had bowel involvement. In the multivariable logistic regression, after adjusting for age and number of cesarean deliveries, body weight was independently associated with a lower likelihood of bowel lesions (odds ratio [OR]: 0.97; 95% confidence interval [CI]: 0.94–0.99; *P* = 0.031). The number of cesarean deliveries was also inversely associated with the outcome (OR: 0.39; 95% CI: 0.21–0.71; *P* = 0.002).

**Conclusion:**

Although higher body weight was associated with a lower chance of bowel involvement, these results should be interpreted cautiously given the cross‐sectional nature of the study and the limitations of BMI and body weight as measures of adiposity. Future studies incorporating more precise anthropometric assessments (e.g., bioimpedance, body fat percentage, or abdominal circumference) are warranted.

## 1 INTRODUCTION

Endometriosis is a chronic, estrogen‐dependent gynecological condition. It is estimated to affect 6%–11% of women of reproductive age.^1^

The most widely accepted pathophysiological theory is retrograde menstruation, in which endometrial fragments reach the peritoneal cavity during menstruation. Other theories include the dissemination of endometrial cells through the lymphatic and hematogenous systems, the coelomic metaplasia theory, and the theory of residual Müllerian embryonic remnants.^2^ Genetic and epigenetic factors have also been implicated.^3^

Several risk factors have been associated with the development of endometriosis, including early menarche, short menstrual cycles, nulliparity, heavy uterine bleeding, and a positive family history.^2^ Factors such as multiparity, hormonal contraceptive use, and obesity are frequently described as potential protective factors.^4^

Obesity is a multifactorial chronic inflammatory condition with increasing prevalence worldwide. In 2020, an estimated 2.6 billion people were living with overweight or obesity, and projections indicate that this number could reach 4 billion by 2035.^5^ Obesity is associated with hormonal and metabolic alterations that include increased peripheral conversion of androgens to estrogen and elevated circulating inflammatory cytokines, such as tumor necrosis factor‐α and interleukin‐6.^6^

These mechanisms could, in theory, contribute to the onset or progression of endometriosis. Nonetheless, population studies indicate an inverse association between body mass index (BMI) and the severity of the disease,^7^ a hypothesis that remains under debate. Proposed explanations include oligomenorrhea or amenorrhea in obese women, leading to reduced retrograde menstrual flow, as well as increased intra‐abdominal pressure, which could mechanically impede the retrograde migration of endometrial tissue through the fallopian tube.^8^

It is estimated that approximately 20% of women with endometriosis develop deep endometriosis.^9^ The intestinal form, particularly involvement of the rectosigmoid, occurs in approximately 12% of cases and poses a therapeutic challenge due to its association with severe pain, functional bowel disturbances, increased surgical morbidity, and a negative impact on quality of life.^10^

Although the relationship between BMI and endometriosis has been examined in several studies, few have specifically investigated the association between obesity and the intestinal form of the disease.

Given the scarcity of data on the prevalence of intestinal endometriosis in women according to body mass index, and the existing controversies regarding the role of obesity in disease severity, this study aims to evaluate the association between obesity and surgically confirmed intestinal endometriosis, seeking to contribute to a better understanding of the clinical pattern of the disease in this context.

## 2 MATERIALS AND METHODS

This was a cross‐sectional observational study involving a retrospective analysis of clinical and surgical data from patients treated at a specialized endometriosis clinic of a tertiary university hospital. Data collection was performed through a review of electronic medical records and surgical protocols previously completed by a specialized medical team.

Women with imaging diagnosis of deep endometriosis (magnetic resonance imaging [MRI] and/or transvaginal ultrasonography with bowel prep) who underwent elective surgery between September 2023 and December 2024 were included. Surgical indication was based on persistent pelvic pain or other symptoms refractory to medical therapy or on evidence of deep organ compromise such as bowel, ureteral, bladder, vascular, or neural involvement. Inclusion criteria comprised: age between 18 and 50 years, histologic diagnosis of deep endometriosis, complete weight (in kilograms) and height (in meters) data, and detailed surgical descriptions specifying the anatomical location of lesions. Patients with chronic inflammatory bowel diseases (e.g., Crohn's disease or ulcerative colitis), untreated hypothyroidism, or Cushing's syndrome were excluded.

The primary outcome was the presence of intestinal endometriosis, defined as a nodular lesion greater than 1 cm in diameter in the rectosigmoid segment, identified intraoperatively and confirmed by histopathology. The main exposure variables were body weight, measured with a calibrated hospital scale during the preoperative visit, and height, measured with a standard stadiometer. BMI was calculated using the formula weight (kg) divided by height squared (m^2^) and categorized according to World Health Organization criteria: BMI <18.5 (underweight), 18.5–24.9 (normal), 25.0–29.9 (overweight), and ≥30.0 (obese).

To characterize the sample, sociodemographic variables were included (age, body weight, BMI, ethnicity, and marital status), along with presence of intestinal lesions, age at menarche, number of vaginal deliveries, cesarean deliveries, and miscarriages, pelvic pain intensity, and duration of pain symptoms (in months).

Statistical analysis was conducted using R software, version 4.5.0. Normality of continuous variables was assessed with the Shapiro–Wilk test. Categorical variables were reported as absolute and relative frequencies, whereas continuous variables were presented as the mean and standard deviation or as the median and interquartile range, depending on data distribution. For comparisons of numerical scores between two independent groups, Student's *t*‐test or the Mann–Whitney test was applied, according to normality assumptions. The association between obesity and presence of intestinal lesions was evaluated using multivariable logistic regression, adjusting for potential confounders. Obesity was represented by BMI or body weight. The outcome variable (intestinal lesion >1 cm) was treated as binary (presence or absence of rectosigmoid involvement). Odds ratios (ORs) were estimated with corresponding 95% confidence intervals (CIs) and *P*‐values. *P*‐values < 0.05 were considered statistically significant.

Multivariable logistic regression was performed in two steps. In the first step, body weight and BMI were included as exposure variables of interest and were entered simultaneously in the same model. Covariates were selected based on clinical rationale (age, age at menarche, number of cesarean deliveries, vaginal deliveries, miscarriages, duration of pain symptoms, ethnicity, and marital status), and only those with *P* < 0.10 in this initial model were retained. In the second step, the variables selected in step 1 were entered into the final model, from which the reported effect sizes (final model coefficients) were obtained. Model adequacy was assessed using variance inflation factor (VIF)‐based multicollinearity diagnostics, residual evaluation, and goodness‐of‐fit testing.

To estimate the predicted probability of the outcome (intestinal endometriosis) as a function of continuous variables that remained significant in the logistic model, we used the estimated marginal means approach. This method calculates adjusted probabilities for specific values of the exposure variable (body weight), while holding the remaining covariates at their mean values. Calculations were performed at three points of each variable: the mean, the mean minus one standard deviation (−1 SD), and the mean plus one standard deviation (+1 SD), on the response scale (probability).

As there are no published studies estimating the effect size (odds ratio) between obesity and intestinal endometriosis and no pilot data were available, sample size assumptions were based on historical data from our endometriosis referral center, where rectosigmoid involvement is approximately 30%. We therefore hypothesized a clinically relevant difference compatible with this overall prevalence, assuming a 10% higher prevalence in women with BMI <25 kg/m^2^ (40%) and a 10% lower prevalence in women with BMI ≥25 kg/m^2^ (20%).

For a comparison of two independent proportions, we adopted a significance level of 5% (*α* = 0.05), statistical power of 80% (1–β = 0.80), a two‐tailed test, and an allocation ratio of 2:1 (two participants with BMI ≥25 for every participant with BMI <25). Based on these parameters, the calculation indicated the need to include 60 participants in the BMI <25 kg/m^2^ group and 120 in the BMI ≥25 kg/m^2^ group, totaling 180 individuals. Anticipating a 15% loss rate, the final sample size was adjusted to 71 participants with BMI <25 kg/m^2^ and 141 with BMI ≥25 kg/m^2^, totaling 212 women to be recruited.

The study was approved by the institution's Research Ethics Committee (protocol number 3725458), with a waiver of informed consent due to its retrospective design and exclusive use of anonymized secondary data.

## 3 RESULTS

The sample consisted of 223 women with surgical and histologic diagnoses of deep endometriosis. The mean age was 38.2 years. Regarding the presence of intestinal nodules, a prevalence of 24.2% (*n* = 54) was observed, whereas 75.8% of patients (*n* = 169) had no intestinal involvement.

Most participants self‐identified as mixed race (48.6%), followed by White (26.6%) and Black women (24.8%). In terms of marital status, 58.7% were in a stable union and 41.3% were single. Table 1 provides detailed characteristics of the study variables. Table 2 presents the demographic and clinical variables stratified by the presence or absence of intestinal nodules.

**TABLE 1:** Demographic and clinical characteristics of the participants.

Characteristic	*N*	*N* = 223^a^
Age (years)	(223)	38.2 ± 6.5
Weight (kg)	(223)	75.0 ± 15.2
BMI	(221)	28.5 ± 5.6
*BMI category*	(221)	
Normal		71 (32)
Obesity		150 (68)
Pain duration (months)	(161)	48 [0, 420]
Menarche	(131)	12.7 ± 1.7
*Vaginal deliveries*	(159)	
0		107 (67)
1		29 (18)
2		14 (8.8)
≥3		9 (5.7)
*Cesareans*	(155)	
0		89 (57)
1		35 (23)
2		24 (15)
3		7 (5)
*Miscarriages*	(150)	
0		125 (83)
1		18 (12)
2		7 (5)
Pelvic pain score	(219)	7.2 ± 2.8
*Intestinal nodule*	(223)	
No		169 (76)
Yes		54 (24)
*Ethnicity*	(218)	
Black		54 (25)
Brown		106 (49)
White		58 (27)
*Marital status*	(213)	
Single		88 (41)
Stable Union		125 (9)

Abbreviation: BMI, body mass index.

^a^ Mean ± standard deviation; *n* (%); median [min, max].

**TABLE 2:** Clinical and demographic characteristics by intestinal nodule.

Characteristic	*N*	Intestinal nodule		*P*‐value^a^
		No *N* = 169^b^	Yes *N* = 54^b^	
Age (years)	223	38.3 ± 6.7	38.1 ± 6.0	0.77
Weight (kg)	223	76.1 ± 15.3	71.6 ± 14.5	0.056
BMI	221	28.8 ± 5.7	27.7 ± 5.1	0.35
*BMI category*	221			0.22
Normal		50 (30)	21 (39)	
Obesity		117 (70)	33 (61)	
Pain duration (months)	161	48 [0, 420]	55 [0, 372]	0.67
Menarche	131	12.6 ± 1.8	12.7 ± 1.6	0.94
*Vaginal deliveries*	159			0.59
0		78 (67)	29 (67)	
1		19 (16)	10 (23)	
2		11 (9.5)	3 (7.0)	
≥3		8 (6.9)	1 (2.3)	
*Cesareans*	155			0.009
0		57 (50)	32 (78)	
1		28 (25)	7 (17)	
2		22 (19)	2 (5)	
3		7 (6)	0 (0)	
*Miscarriages*	150			0.32
0		88 (81)	37 (88)	
1		13 (12)	5 (12)	
2		7 (6)	0 (0)	
Pelvic pain score	219	7.2 ± 2.8	7.1 ± 2.8	0.95
*Ethnicity*	218			0.67
Black		40 (24)	14 (26)	
Brown		83 (50)	23 (43)	
White		42 (25)	16 (30)	
*Marital status*	213			0.41
Single		64 (40)	24 (46)	
Stable Union		97 (60)	28 (54)	

^a^ Wilcoxon rank sum test; Pearson's *χ* ^2^‐test; Fisher's exact test.

^b^ Mean ± standard deviation; *n* (%); median [min, max].

In the initial multivariable logistic regression model (first step), both BMI and body weight were tested as the main exposure variables. Even with both variables included simultaneously, the VIF was 2.3, indicating no meaningful multicollinearity. BMI was not statistically significant (*P* = 0.37).

In step 1, body weight (*P* = 0.025), number of previous cesarean deliveries (*P* = 0.005), and age (*P* = 0.005) were selected (*P* < 0.10) and entered the final model (step 2), which was used to estimate effect sizes. In the step 2 (final model) multivariable logistic regression model (Table 3), body weight remained independently associated with a lower likelihood of intestinal involvement. Specifically, for each 1‐kg increase in body weight, there was an approximate 3% reduction in the odds of presenting an intestinal lesion (OR: 0.97; 95% CI: 0.94–0.99; *P* = 0.031). The number of prior cesarean deliveries was also associated with a lower probability of intestinal disease (OR: 0.39; 95% CI: 0.21–0.71; *P* = 0.002). Age demonstrated a positive association (OR: 1.05; 95% CI: 0.99–1.12; *P* = 0.115), although it did not reach statistical significance.

**TABLE 3:** Multiple logistic regression for intestinal nodule.

Characteristic	OR	95% CI	*p*‐value
Age (years)	1.05	0.99–1.12	0.118
Weight (kg)	0.97	0.94–0.99	0.031
Cesarean	0.39	0.21–0.71	0.002

Abbreviations: CI, confidence interval; OR, odds ratio.

Estimated marginal means showed an inverse association between body weight and the predicted probability of intestinal lesions. The predicted probability was 31.7% for women with body weight corresponding to the mean minus one standard deviation (59.8 kg; 95% CI: 21.0–44.6), decreasing to 22.4% at the mean (74.7 kg; 95% CI: 15.7–30.8) and to 15.2% at the mean plus one standard deviation (89.5 kg; 95% CI: 8.4–25.9) (Table 4). These 95% confidence intervals refer to the absolute predicted probability (percentage points); that is, for 59.8 kg, the predicted probability is estimated to range from 21.0% to 44.6%.

**TABLE 4:** Estimated marginal means for the probability of intestinal endometriosis stratified by the corporal weight.

Weight	Probability	SE	95% confidence interval	
			Lower	Upper
59.800^−^	0.317	0.0611	0.2104	0.4460
74.700^μ^	0.224	0.0386	0.1570	0.3080
89.500^+^	0.152	0.0440	0.0840	0.2590

*Note*: −, mean – 1 SD; μ, mean; +, mean + 1 SD.

Abbreviations: SD, standard deviation; SE, standard error.

## 4 DISCUSSION AND CONCLUSION

In this retrospective cross‐sectional study, we observed that women with higher body weight had a lower prevalence of surgically and histologically confirmed rectosigmoid intestinal lesions. We considered only nodules larger than 1 cm at the time of surgery as true intestinal disease to minimize false‐positive classifications. After adjustment for age and number of previous cesarean deliveries, body weight remained negatively associated with intestinal involvement (OR 0.97; 95% CI: 0.94–0.99), indicating a significant reduction of approximately 3% in the odds of intestinal disease for each additional kilogram. Although this per‐kilogram effect is modest, it may still be clinically meaningful across larger differences in body weight: for example, a 10 kg difference would correspond to an approximate 26% lower odds of intestinal involvement (OR = 0.74). This observation aligns with previous reports suggesting that obesity might, paradoxically, exert a protective role against anatomically more severe forms of endometriosis.^1^, ^11^

These findings reinforce the hypothesis that excess body weight might exert a mechanical protective effect against disease progression.^11^ One unproven hypothesis is that adipose tissue in the posterior compartment might act as an anatomic barrier, hindering disease progression, particularly toward the rectosigmoid. Future studies could test this potential mechanical protective effect by combining imaging‐based quantification of posterior compartment fat (e.g., MRI‐derived measurements) with standardized surgical mapping of lesion distribution and depth of infiltration. BMI, like body weight, was negatively associated with intestinal disease, although it did not reach statistical significance in the multivariable model.

It is now recognized that BMI‐based classification alone is not adequate for the individualized assessment of patients with obesity, as it underestimates true visceral and subcutaneous adiposity. Moreover, BMI does not distinguish between fat mass and lean mass, which might overestimate its clinical relevance and lead to misclassification.^12^

Body mass index values above 40 (morbid obesity) are considered a more acceptable parameter when used as an isolated factor for assessing obesity.^12^ However, because no patients with this profile were identified in our sample, additional studies are needed to confirm this hypothesis, preferably using other anthropometric measures capable of more accurately defining clinical obesity, such as abdominal circumference and bioimpedance analysis.

The relationship between obesity and endometriosis has been a subject of controversy in literature. On one hand, adipose tissue is an important source of peripheral estrogens, particularly through the conversion of androgens to estrone via aromatase,^6^ which could theoretically stimulate the progression of endometriotic lesions. On the other hand, women who are overweight or obese tend to exhibit anovulation, oligomenorrhea, or amenorrhea—conditions that reduce the number of menstrual cycles and, consequently, the exposure to retrograde flow, one of the most widely accepted pathophysiological theories for the development of endometriosis. In addition, mechanical factors, such as increased intra‐abdominal pressure, may act as a physical barrier to tubal reflux, limiting the implantation of endometrial cells in the pelvic cavity.^8^ Another mechanical factor highlighted by this study relates to the hypothesis that a protective layer of adipose tissue may hinder infiltration into retroperitoneal structures.

The present finding of lower intestinal involvement among patients with higher body weight reinforces these hypotheses, particularly regarding deep intestinal endometriosis, one of the most complex and clinically significant forms of the disease. This specific phenotype is associated with high morbidity, greater need for bowel resections, and a more pronounced impact on quality of life,^10^ underscoring the importance of understanding its predisposing or protective factors.

Moreover, the number of previous cesarean deliveries was also inversely associated with the presence of intestinal lesions, with an adjusted OR of 0.39 (95% CI: 0.21–0.71). Although a causal relationship cannot be established, women with a higher number of cesarean deliveries might be considered more fertile, serving as a potential marker of less severe disease, given that pregnancy might attenuate disease progression, as might the duration of breastfeeding—an aspect not investigated in the present study. The number of vaginal deliveries was likewise negatively associated with intestinal disease, although it did not reach statistical significance in the multivariable model. One hypothesis is that cesarean delivery might be related to postoperative adhesions capable of altering pelvic anatomy and, theoretically, hindering the implantation of lesions in the posterior compartment.

Although these explanations are plausible, they remain unproven and should be interpreted cautiously. It is also worth noting that women with endometriosis have a higher risk of cesarean delivery, whether in spontaneous or assisted (in vitro fertilization) pregnancies.^13^, ^14^ Obese women also present an increased risk of cesarean delivery due to the higher likelihood of preeclampsia and diabetes, intrauterine growth restriction, polyhydramnios, acute fetal distress, among other complications.^15^ Therefore, the association between obesity and endometriosis, even when not specifically intestinal, would be expected to increase the prevalence of cesarean delivery.

Age did not show a statistically significant association with bowel endometriosis in the final model (*P* = 0.11). However, previous studies have reported that older women tend to present more advanced stages of endometriosis.^16^ This pattern likely reflects the longer time available for disease progression before diagnosis, considering that endometriosis might continue to progress over the years even with appropriate treatment.

This study has several limitations, including its cross‐sectional design, which prevents causal inference due to issues of temporality and potential residual confounding. In addition, the use of body weight as a continuous variable, although statistically robust, does not capture the complexity of body fat distribution (the same limitation applies to BMI), which might be metabolically more relevant than total weight. Variables such as abdominal circumference or body fat percentage measured through more precise techniques should be considered in future studies.

Despite these limitations, this study contributes to the understanding of the relationship between obesity and deep intestinal endometriosis, highlighting the importance of individualized and multidimensional evaluation of patients with endometriosis. Future research with prospective study designs that incorporate more accurate anthropometric measurements is essential to better elucidate this relationship.

Although the findings suggest a possible protective role of higher body weight against the intestinal form of endometriosis, this should not be interpreted as indicating that obesity is beneficial. The management of obesity should remain a clinical priority, both because of its associated metabolic risks and its potential impact on pain intensity, psychological health, and response to hormonal therapy.

## AUTHOR CONTRIBUTIONS

**Tereza Carolina Fonseca Corrêa:** Data curation (lead), writing—original draft preparation (lead), writing—review and editing (equal), visualization (equal) conceptualization (equal), investigation (lead), methodology (equal). **Marco Aurelio Pinho Oliveira:** Writing—review and editing (lead), validation (lead), supervision (lead), visualization (equal), methodology (equal), formal analysis (lead), software (lead). All authors were directly involved in the statistical analysis, data collection, figure preparation, and writing.

## FUNDING INFORMATION

The authors have nothing to report.

## CONFLICT OF INTEREST STATEMENT

The authors have no conflicts of interest.

## Data Availability

Data available on request from the authors.

## References

[1] Rowlands IJ , Hockey R , Abbott JA , Montgomery GW , Mishra GD . Body mass index and the diagnosis of endometriosis: findings from a national data linkage cohort study. Obes Res Clin Pract. 2022;16(3):235‐241. 35431154 10.1016/j.orcp.2022.04.002

[2] Kvaskoff M , Mu F , Terry KL , et al. Endometriosis: a high‐risk population for major chronic diseases? Hum Reprod Update. 2015;21(4):500‐516. 25765863 10.1093/humupd/dmv013 PMC4463000

[3] Koninckx PR , Ussia A , Adamyan L , Wattiez A , Gomel V , Martin DC . Pathogenesis of endometriosis: the genetic/epigenetic theory. Fertil Steril. 2019;111(2):327‐340. 30527836 10.1016/j.fertnstert.2018.10.013

[4] Hemmings R , Rivard M , Olive DL , et al. Evaluation of risk factors associated with endometriosis. Fertil Steril. 2004;81(6):1513‐1521. 15193470 10.1016/j.fertnstert.2003.10.038

[5] Janić M , Janež A , El‐Tanani M , Rizzo M . Obesity: recent advances and future perspectives. Biomedicine. 2025;13(2):368. 10.3390/biomedicines13020368 PMC11853004 40002780

[6] Heymsfield SB , Wadden TA . Mechanisms, pathophysiology, and Management of Obesity. N Engl J Med. 2017;376(3):254‐266. 28099824 10.1056/NEJMra1514009

[7] Holdsworth‐Carson SJ , Dior UP , Colgrave EM , et al. The association of body mass index with endometriosis and disease severity in women with pain. J Endometr Pelvic Pain Disord. 2018;10(2):79‐87.

[8] Zondervan KT , Becker CM , Koga K , Missmer SA , Taylor RN , Viganò P . Endometriosis. Nat Rev Dis Primers. 2018;4:9. 30026507 10.1038/s41572-018-0008-5

[9] Widschwendter P , Köhler M , Friedl T , et al. Diagnosis of presence and extent of deep infiltrating endometriosis by preoperative MRI‐improvement of staging accuracy by expert training. J Gynecol Obstet Hum Reprod. 2022;51(1):102236. 34592437 10.1016/j.jogoh.2021.102236

[10] O'Brien L , Morarasu S , Morarasu BC , et al. Conservative surgery versus colorectal resection for endometriosis with rectal involvement: a systematic review and meta‐analysis of surgical and long‐term outcomes. Int J Color Dis. 2023;38(1):55. 10.1007/s00384-023-04352-6 36847868

[11] Pantelis A , Machairiotis N , Lapatsanis DP . The formidable yet unresolved interplay between endometriosis and obesity. Sci World J. 2021;2021:1‐10. 10.1155/2021/6653677 PMC8079185 33986637

[12] Rubino F , Cummings DE , Eckel RH , et al. Definition and diagnostic criteria of clinical obesity. Lancet Diabetes Endocrinol. 2025;13(3):221‐262. 39824205 10.1016/S2213-8587(24)00316-4 PMC11870235

[13] Vercellini P , Viganò P , Bandini V , Buggio L , Berlanda N , Somigliana E . Association of endometriosis and adenomyosis with pregnancy and infertility. Fertil Steril. 2023;119(5):727‐740. 36948440 10.1016/j.fertnstert.2023.03.018

[14] Breintoft K , Pinnerup R , Henriksen TB , et al. Endometriosis and risk of adverse pregnancy outcome: a systematic review and meta‐analysis. J Clin Med. 2021;10(4):667. 33572322 10.3390/jcm10040667 PMC7916165

[15] Vendittelli F , Barasinski C , Rivière O , Bourdel N , Fritel X . Endometriosis and risk of adverse pregnancy outcomes: a retrospective multicenter cohort study. Fertil Steril. 2025;123(1):137‐147. 39089610 10.1016/j.fertnstert.2024.07.037

[16] Yang Y , Li J , Chen H , Feng W . Assessment of risk factors associated with severe endometriosis and establishment of preoperative prediction model. Diagnostics (Basel). 2022;12(10):2348. 36292037 10.3390/diagnostics12102348 PMC9601177

